# Exploratory data analysis of a clinical study group: Development of a procedure for exploring multidimensional data

**DOI:** 10.1371/journal.pone.0201950

**Published:** 2018-08-23

**Authors:** Bogumil M. Konopka, Felicja Lwow, Magdalena Owczarz, Łukasz Łaczmański

**Affiliations:** 1 Department of Biomedical Engineering, Faculty of Fundamental Problems of Technology, Wroclaw University of Science and Technology, Wroclaw, Poland; 2 Department of Health Promotion, Faculty of Physiotherapy University School of Physical Education, Wroclaw, Poland; 3 Mossakowski Medical Research Centre, Polish Academy of Sciences, Warsaw, Poland; 4 International Institute of Molecular and Cell Biology, Warsaw, Poland; 5 Hirszfeld Institute of Immunology and Experimental Therapy, Polish Academy of Sciences, Wroclaw, Poland; University of Nebraska Medical Center, UNITED STATES

## Abstract

Thorough knowledge of the structure of analyzed data allows to form detailed scientific hypotheses and research questions. The structure of data can be revealed with methods for exploratory data analysis. Due to multitude of available methods, selecting those which will work together well and facilitate data interpretation is not an easy task. In this work we present a well fitted set of tools for a complete exploratory analysis of a clinical dataset and perform a case study analysis on a set of 515 patients. The proposed procedure comprises several steps: 1) robust data normalization, 2) outlier detection with Mahalanobis (MD) and robust Mahalanobis distances (rMD), 3) hierarchical clustering with Ward’s algorithm, 4) Principal Component Analysis with biplot vectors. The analyzed set comprised elderly patients that participated in the PolSenior project. Each patient was characterized by over 40 biochemical and socio-geographical attributes. Introductory analysis showed that the case-study dataset comprises two clusters separated along the axis of sex hormone attributes. Further analysis was carried out separately for male and female patients. The most optimal partitioning in the male set resulted in five subgroups. Two of them were related to diseased patients: 1) diabetes and 2) hypogonadism patients. Analysis of the female set suggested that it was more homogeneous than the male dataset. No evidence of pathological patient subgroups was found. In the study we showed that outlier detection with MD and rMD allows not only to identify outliers, but can also assess the heterogeneity of a dataset. The case study proved that our procedure is well suited for identification and visualization of biologically meaningful patient subgroups.

## Introduction

Thorough knowledge of the structure of analyzed data allows to form detailed scientific hypotheses and research questions. It is crucial for correct interpretation of conducted experiments. This is especially important in case of investigations where the researcher does not directly control the conditions or the investigated objects. Clinical or epidemiological studies can be examples of such investigations. Here we will present a case-study analysis of a group of 515 elderly participants of an epidemiological study. Despite the fact that usually participants of clinical studies go through a qualification procedure, fill in detailed question forms and need to meet requirements regarding biochemical parameters, age, health history etc., it may happen that a gathered dataset still contains individuals that should not take part in the study. Their presence in the dataset may significantly influence its final outcome and lead to false conclusions.

The data structure and basic associations between parameters in the data can be revealed with methods for exploratory data analysis, such as clustering or Principal Component Analysis (PCA). Distanced based data analysis methods (including many types of clustering and PCA) are sensitive to data scaling. Therefore data normalization is often needed. Typically this can be performed with Z-score normalization, which assumes normal distribution of values of an attribute. It indicates how many standard deviations an instance of the data is away from the sample mean. Another often used normalization method is the Min-max normalization, which scales an attribute to a 0–1 range. It is especially useful when the bottom and top values of the attribute are limited—for instance due to experimental design. These normalization techniques are sensitive to outliers. The robust Z-score normalization is a modification of the classic Z-score normalization in which median is used instead of the mean and interquartile range is used instead of the standard deviation. These changes minimize the influence of extreme values on the resulting normalization.

Identification of outliers in the data set is another important step in the analysis. Outliers are instances of data that are characterized by extreme attribute values in comparison to the core of the dataset. An outlier can be defined as an instance that was generated by a different process than the rest of instances [1]. Outliers in single dimensional data can be filtered out with univariate statistic based methods [2]. However, for high-dimensional data more sophisticated methods need to be used. These methods can be divided into 1) model-based approaches, which assume a model of data—if a data point does not fit the model, it is labelled as an outlier [3], [4], 2) proximity-based approaches, which calculate the distance between a data point and all other data—outliers are points that show significantly different distances [5], [6] 3) angle-based approaches, which calculate the angles between a data point and all other data, outliers are points that acquire small fluctuations of angles [7]. Thorough reviews of outlier detection techniques can be found in [8], [9] and [10].

The structure of pre-processed data can be investigated with clustering techniques. These fall into several main categories: 1) hierarchical clustering, 2) partitioning relocation methods (which include various versions of K-means and K-medoids), 3) density-based partitioning, and 4) grid-based partitioning, which performs segmentation of attribute space and agglomeration of similar segments. For a review see [11]. Among these, hierarchical clustering is associated with probably the clearest way of visualization, i.e. the dendrogram also called the clustering tree, which allows detailed investigation of every clustering step. That is why it is especially useful in data exploration. Clustering quality can be verified quantitatively with clustering validation indices, such as Dunn index [12], Davies-Bouildin index [13] or silhouette values [14].

Data visualization is an extremely important element of data exploration analysis. It allows to connect facts and form conclusions based on the outcome of other steps of the analysis. A classical method for visualization of multidimensional data is PCA [15], which allows to reduce the number of dimensions needed to depict a dataset without a significant loss of information. However this can also be performed with multidimensional scaling [16] or some other nonlinear dimensionality reduction techniques [17].

As it can be seen from this short introduction, when facing the problem of getting to know a new dataset, a researcher has a plethora of exploratory tools to choose from. Selecting methods that will work together and facilitate revealing the structure of the data is not an easy task. In this work we present a well fitted set of tools for a complete exploratory analysis of a clinical study dataset. We perform a case-study analysis in which we address the most important questions that need to be asked prior to most studies: are there any significant outliers in the dataset? What subgroups make up for the dataset? What are the characteristics of particular subgroups? And finally, what are the biological reasons that underlie such dataset structure?

## Methods

### Dataset description

The presented analysis is part of a project which aims at investigating the relation between certain polymorphisms of a gene–Vitamin D Receptor and sex hormone levels in elderly people. The research sample was chosen from the PolSenior study [18]—a project that aims at investigating the interrelations between health, genetics and social status in advanced age in Polish population.

The dataset consisted of 515 participants– 238 women, and 277 men, whose age was in the range 55–102 years. Each participant was described by 23 numeric and 21 nominal attributes (S1 Table). Numeric attributes contain biophysical and biochemical parameters, such as AGE, WEIGHT and BLOOD INSULIN CONCENTRATION. Nominal attributes include socio-geographical data such as COUNTRY REGION, CITY POPULATION, and also SEASON and MONTH. The full list of attributes and their description is given in Tables 1 and 2. The study was approved by Bioethical Committee of the Medical University of Silesia (KNW-6501-38/I/08) and informed written consent, including consent for genetic studies, was obtained from all of the subjects before testing.

**Table 1:** Patient numerical attributes. Most are biochemical parameters.

Attribute name	Description
AGE	age in years
HEIGHT	height given in [cm]
WEIGHT	weight in [kg]
WAISTLINE	waistline given in [cm]
HIP.GIRTH	hip girth given in [cm]
BMI	the body to mass index [kg/m^2^]
FAT	Amount of body fat as percentage of body weight [%]
CHOL.HDL	Cholesterol serum level—High Density Lipoprotein [mg/dl]
CHOL.LDL	cholesterol serum level—Ligh Density Lipoprotein [mg/dl]
CHOL.TOTAL	total level of cholesterol [mg/dl]
TGC	serum level of triglycerides [mg/dl]
GLUCOSE	Serum Glucose level [mg/dl]
INS	serum level of insulin [μIU/ml]
TESTOSTERONE	serum level of testosterone [nmol/l]
ESTRADIOL	serum level of Estradiol [pmol/l]
DHEA.S	serum level of Dehydroepiandrosteron [ng/dl]
SHGB	serum level of sex hormone binding globulin [pmol/l]
FAI	Free Androgen Index defined as the ratio of total testosterone to SHBG × 100 [19]
FEI	Free Estradiol Index defined as the ratio of total estradiol to SHBG × 100 [19]
FSH	Serum Follicle-Stimulating Hormone level [IU/l]
ICTP	serum level of carboxy-terminal cross-linked telopeptide of type I collagen [mg/l]
OPG	serum level of osteoprotegerin [pmol/l]
VITAMIN.D	serum level of Vitamin D [ng/ml]

**Table 2:** Patient categorical and nominal attributes.

Attribute name	Description
AGE.GROUP	age in discretized groups (5 year bins)
CG1.IDENTIFIED.DIABETES.YES	binary; 1 if observed
CITY.SIZE	city size bins: countryside, population < 20 thousand, 20–50 thousand, 50–200 thousand, 200–500 thousand, >500 thousand
HYPERANDROGENISM.YES	binary; 1 if observed
HYPERTENSION.YES	binary; 1 if observed
INSOLATION.YES	binary; 1 if in summer and spring
MACROREGION	6 binary attributes: ‘north’, ‘east’, ‘south’, ‘central’, ‘north-west’, ‘south-west’
OBESTIY_PHENO_FLMHO	binary; obesity phenotype—metabolic healthy obesity [20]
OBESTIY_PHENO_FLMONW	binary; obesity phenotype—methabolic obesity normal weight [20]
OBESTIY_PHENO_FLOMWD	binary; obesity phenotype—obesity methabolic weist disease [20]
OBSETITY_PHENOOBZM	binary; obesity phenotype–adjustment of FLMHO for Polish population [20]
OBSETITY_PHENOOZZM	binary; obesity phenotype–adjustment of FLMONW for Polish population [20]
YEAR_SEASON	4 binary attributes: ‘winter’, ‘spring’, ‘summer’, ‘autumn’

### Data exploration procedure

As mentioned in the introduction: data visualization and clustering are crucial for understanding the data at hand. These were key elements of the procedure proposed in the study. In order to visualize multidimensional data in a two dimensional space, dimension reduction has to be performed. We used PCA which is a classical method, available in most statistical packages. Using PCA requires data scaling, otherwise attributes with highest variance may dominate the outcome. For the same reason outliers need to be detected and removed.

The exploratory analysis was carried out in two stages. First, we conducted the exploratory analysis based on numeric attributes (Table 1) using the following procedure: 1) normalization, 2) Principal Component Analysis, 3) Outlier detection and removal, 4) clustering. After that, clustering was repeated with the nominal/categorical attributes added (Table 2). We performed the analysis in two stages because processing numerical data is more straightforward–most analysis algorithms were designed to treat numerical data. Processing nominal data requires additional actions to transform from the nominal attribute space to a numerical one and the results need to be analyzed with great caution.

### Normalization

All numerical attributes were normalized using Robust Z-Score Normalization (Eq 1):

$$x_{normalized}=\frac{x-median(x)}{IQR(x)},$$ (1)

where IQR(x) is the interquartile range of the attribute. Applying Robust Z-score Normalization insures that the influence of any potential outliers on the normalization is minimal.

### Principal Component Analysis (PCA)

Basic R package function ***prcomp*** was used for calculation of principal components (PCs). The PC ***biplot*** was used for visualization of PCs along with variability and contributions of original attributes [21]. PCA was carried out on normalized data.

### Outlier detection

Two approaches were used to detect outlying samples.

The Mahalanobis Distance [4] is defined as:

$$MD(x_{i})=\sqrt{\left(x_{i}-\overset{-}{X})S_{0}^{-1}(x_{i}-\overset{-}{X}\right)},$$ (2)

where ***x***_***i***_ is the vector of attribute values of ***i-th*** sample, $\overset{-}{X}$ is the m-dimensional vector of attribute means and ***S***_**0**_ is the covariance matrix calculated for the whole dataset.

The robust Minimum Covariance Determinant (MCD) is a modification of Mahalanobis distance as defined in [3]. It is also called the robust Mahalanobis Distance (rMD). The MCD algorithm is an iterative procedure. The steps are:

1. Chose a subset ***H*** of size ***h***.
2. Calculate $\overset{-}{X_{1}}$ and ***S***_**1**_ for samples in ***H***
3. Calculate distance ***rMD*(*x***_***i***_**)** for ***i = 1*,..,*n***, with $\overset{-}{X_{1}}$ and ***S***_**1**_ instead of $\overset{-}{X}$ and ***S***_**0**_:
  $$rMD(x_{i})=\sqrt{(x_{i}-\overset{-}{X_{k}})S_{k}^{-1}(x_{i}-\overset{-}{X_{k}}),}$$ (3)
  where k is the iteration number.
4. Sort all samples in terms of *rMD*(*x*_*i*_).
5. Choose a new subset *H*_2_ of ***h*** samples with the smallest ***rMD***.
6. Repeat 1–5 untill ***det***(***S***_***k***_) = **0** or ***det***(***S***_***k***_) = ***det***(***S***_***k***−**1**_), where k is the iteration number.

The intuitive difference between ***MD*** and ***rMD*** is that, in case of ***MD*** outliers influence $\overset{-}{X}$ and ***S***_**0**_ (Eq 3), while in rMD only a subset of ***h*** samples is used for calculating $\overset{-}{X_{k}}$ and ***S***_***k***_ thus the influence of outliers on the calculated distances is limited.

Both MD and rMD were calculated using the ‘chemometrics’ R Package [22].

### Hierarchical clustering analysis

The main clustering approach used was hierarchical clustering. It was performed in two steps. First, samples were clustered based only on numerical attributes. Then, nominal attributes were incorporated for a joined cluster analysis. Nominal attributes were binarized and then rescaled, so that 0 and 1 equaled the I-st and the III-rd quartile of the distribution of all numerical values. This way the center of the data remained unchanged upon addition of nominal attributes. Simultaneously, we performed clustering of attributes. We used hierarchical agglomerative clustering using Ward method, which minimizes the change in variance resulting from fusion of two clusters [23]. Technically, calculations were carried out with *hclust* R function with the “ward.D2” method.

Dunn [12] and Davies–Bouldin [13] indices were used to support this cluster analysis and index proper number of clusters. The indexes were calculated using the ‘clv’ R Package [24].

Dunn index is defined as:

$$Dunn={\text{min}}_{i=1,..,\;nc}\left\{{\text{min}}_{j=i+1,..,nc}\left(\frac{d(c_{i},c_{j})}{{\text{max}}_{k=1,\ldots ,nc}diam(c_{k})}\right)\right\},$$ (4)

where ***nc***, denotes number of clusters, ***c***_***i***_ is the i-th cluster, ***d(c***_***i***_, ***c***_***j***_***)*** is the dissimilarity between clusters ***i*** and ***j***, and ***diam(c)*** is a function used for assessing the dispersion of a cluster.

Davies-Bouldin is calculated as:

$$DB=\frac{1}{nc}\sum_{i=1}^{nc}{\text{max}}_{j}R_{ij},$$ (5)

where ***R***_***ij***_ measures the relations between each pair of clusters defined as:

$$R_{ij}=\frac{diam(c_{i})+diam(c_{j})}{d(c_{i},\;c_{j})},$$ (6)

where ***d(c***_***i***_, ***c***_***j***_***)*** is the dissimilarity between clusters ***i*** and ***j***, and ***diam(c)*** is a function used for assessing the dispersion of a cluster.

During calculation of Dunn and DB indices we chose ***diam(c)*** to be the average distance between cluster members and cluster centroids, and ***d(c***_***i***_, ***c***_***j***_***)*** to be the distance between centroids of compared clusters. The choice was implied by the fact that Ward’s clustering algorithm minimizes the within-cluster variance which is defined as the average distance between cluster members and cluster centroids, and also maximizes the inter-cluster variance which is based on centroid locations [23]. Therefore, such a choice of measures for Dunn and DB gives the best insight into the outcome of clustering.

### Additional cluster analysis

Hierarchical clustering analysis of the male set was additionally supported with three other clustering techniques: 1) density-based DBSCAN clustering [25], 2) clustering based on PCAs and 3) biclustering in order to verify the main conclusions.

Density Based clustering depends on two input parameters, i.e. number of neighbors required to start a new cluster–*K*, and the distance defining the neighborhood of a point–*epsilon*. K was set to 3 based on visual inspection of the dataset, while epsilon was set to 4 based on k Nearest Neighbor Distance plot (see Results). The choice was the y-value beyond which the distances increased rapidly. We used the DBSCAN R package implementation of the algorithm [26].

PCA-based clustering was performed on top 7 PCs, which accounted for 70% of data variance. The same routine as for main hierarchical clustering was used, i.e. euclidean distance and Wards algorithm as implemented in R stats package.

The biclustering approach used was the Plaid Models clustering [27], which allows to identify subsets of rows and columns with coherent values. In case of the analyzed dataset those subsets could be regarded as subgroups of patients presenting similar dependence of particular attributes. The biclust package implementation of the algorithm was used [28].

### Statistical testing

Significance of differences between all clusters in terms of particular attributes was first tested with the Kruskal-Wallis test [29]–h_0_: distributions are the same in all groups. Then paired Wilcoxon rank sum test with Bonferroni correction was used to evaluate the head-to-head difference significance. Both are non-parametric test available in R basic {stats} package.

## Results & discussion

### Introductory analysis

**Fig 1:**
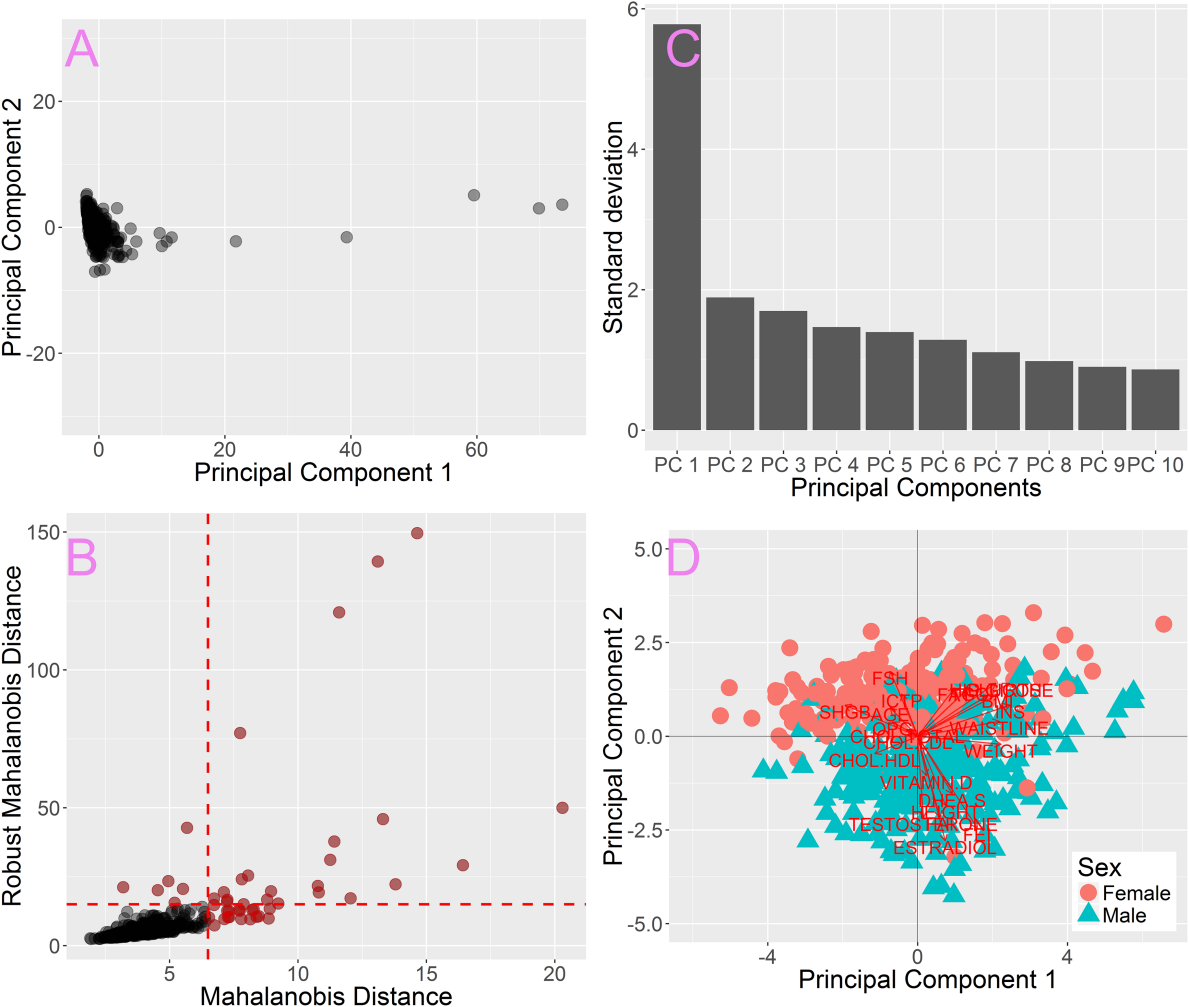
Introductury dataset analysis with PCA. A) PCA carried out on full dataset. B) standard deviations of first 10 PCs indicate that the first PC dominates the variability of the dataset. C) The MD vs rMD plot allows to identify the most distant outliers (red points). D) PCA carried out after removal of most distant samples shows that male and female patients form two distinct clusters.

Firstly, raw data were normalized using the robust Z-score normalization then PCA was carried out. The plot of first two components shows that there are significant outliers in the data set (Fig 1A). The first component clearly dominates the remaining ones (Fig 1B). The main contribution to the first component comes from the INSULINE level (data not shown) due to increased variability caused by outliers. MD vs rMD plot shows that the majority of data forms a core (Fig 1C–grey points) and also confirms the presence of significantly outlying samples (Fig 1C–red points).

In order to get an overall look at the core of data we used arbitrarily set MD and rMD thresholds to remove the most distant outliers, 6.5 and 15 respectively (Fig 1C–dashed lines). The thresholds were selected so that only the core of the data remained.

The plot of two first components, calculated after removing outlying points, reveals that samples are grouped in two clusters, consisting of male and female patients respectively (Fig 1D). The biplot [1] allows to visualize contributions of original attributes to particular PCs in the form of vectors. For instance if a patient had a level of ESTRADIOL higher than average, then in the PCA with biplot vectors he/she would be moved away from the center of the plot in the direction pointed by the ESTRADIOL vector. It can be seen that the two acquired clusters are separated along an axis formed by attributes such as: ESTRADIOL, TESTOSTERONE, FEI, FAI, FSH, which are sex hormones (Fig 1D–red vectors). Such strong separation suggests that further analysis should be carried out separately for male and female patients. The position of particular samples in Fig 1D is also strongly influenced by a group of attributes perpendicular to the sex hormone axis. These attributes are generally related to metabolism: such as GLUCOSE, INSULINE, FAT, WEIGHT etc. The fact that these attributes are perpendicular to the sex hormone axis suggested they are unrelated to patient sex.

### Male set analysis

In the first part of male set analysis all 277 male patients with all 23 numeric attributes from the raw dataset were analyzed. Again robust Z-score normalization was performed.

### Outlier detection

**Fig 2:**
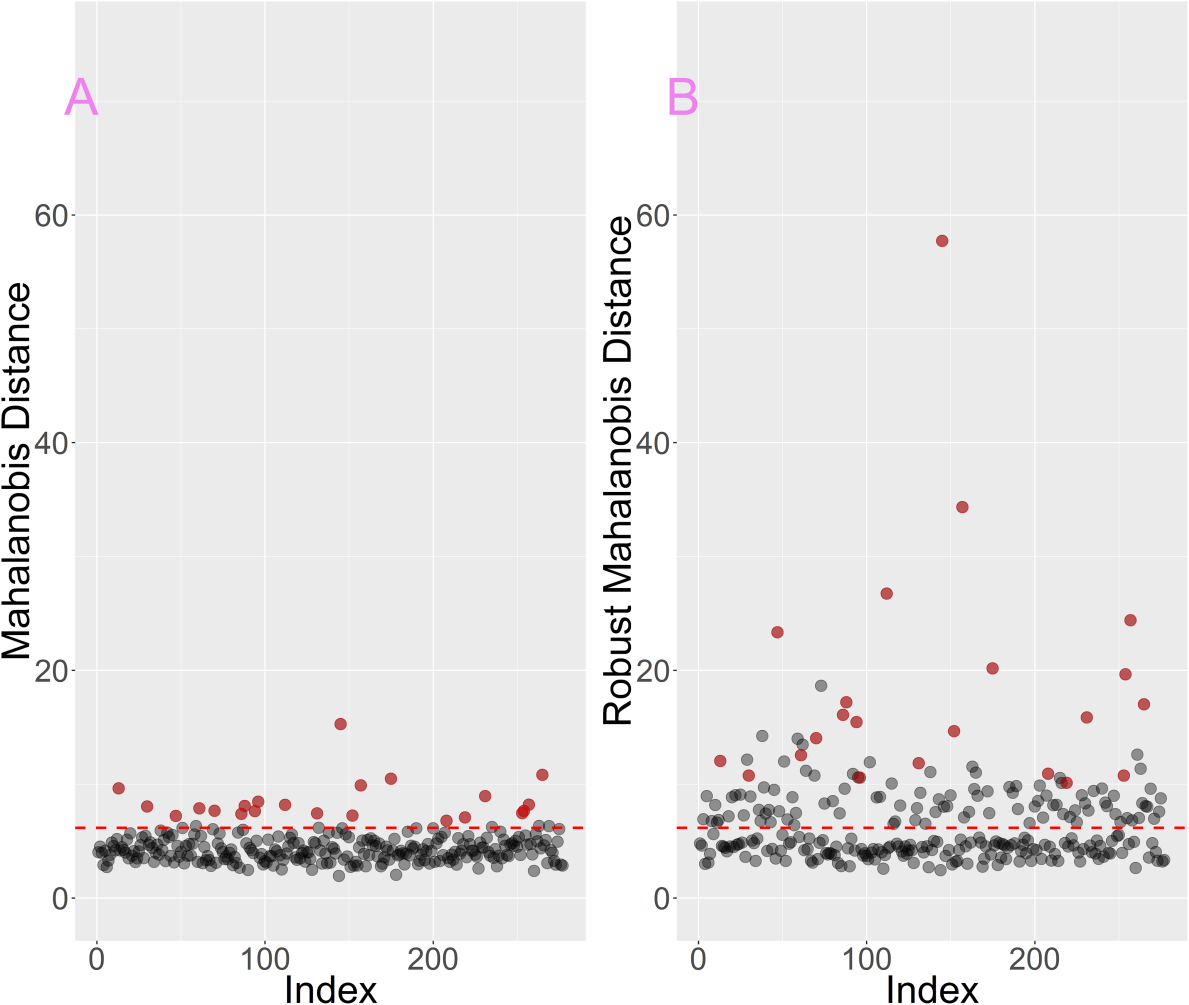
Outliers in the male dataset. A) according to classic MD, B) according to rMD. Outliers according to MD are colored red in both plots. The dashed line denotes the 0.99 quantile threshold for Chi2 distribution used for flagging outliers.

According to MD there are 22 outliers in the dataset. These points clearly stand out in terms of MD values from the rest of the set (Fig 2A–red points). In terms of rMD there are many more candidate outliers, i.e. 124 samples. Both measures are consistent with regard to MD outliers—all samples pointed as outliers by the classic MD were also outliers in terms of rMD, what is more these were among the points with the highest rMD values (Fig 2b–red points). The fact that rMD indicated almost half of the dataset as outliers may suggest that the set is heterogeneous.

**Fig 3:**
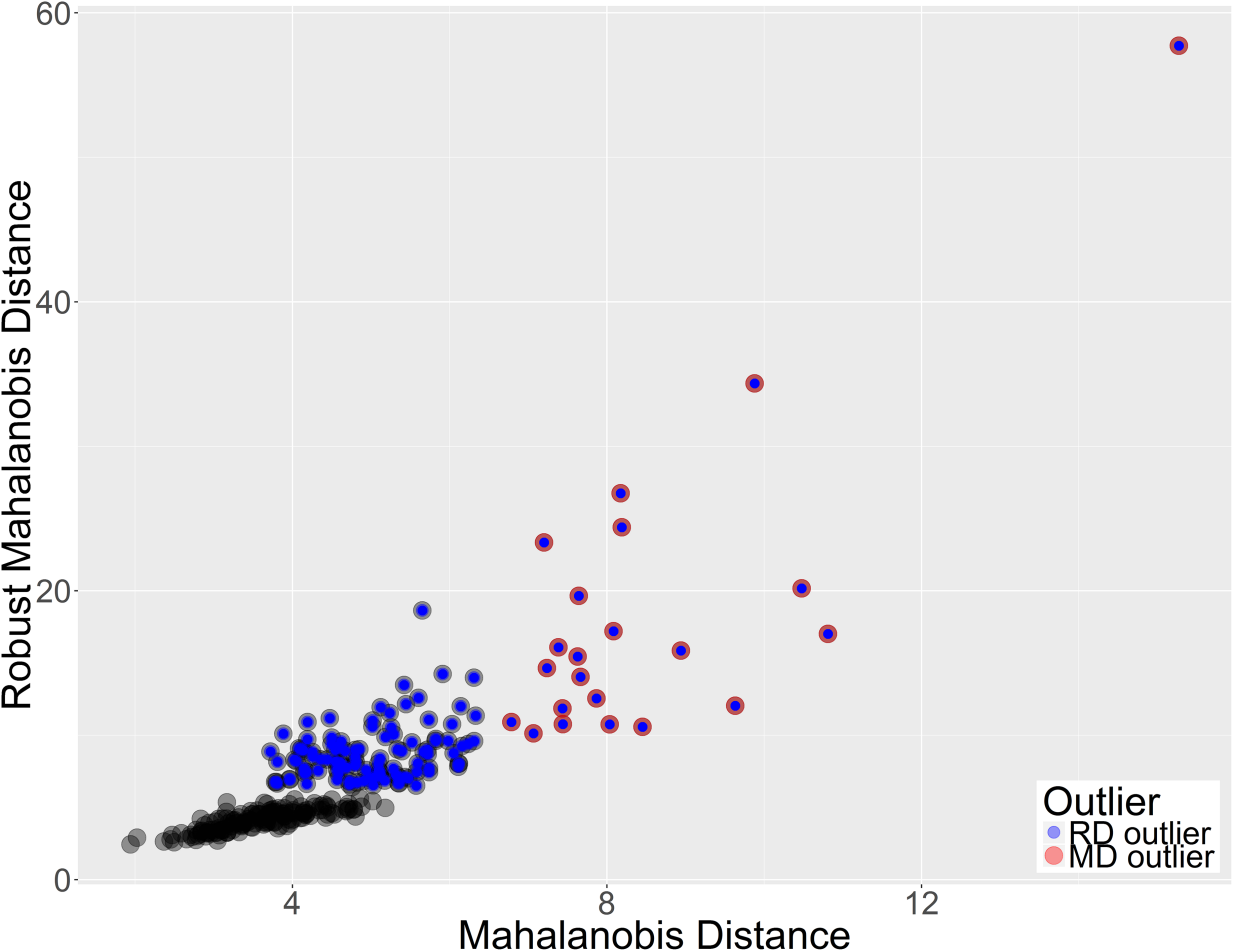
Mahalanobis Distance vs robust Mahalanobis Distances for male dataset. Outliers were marked with blue and red points for rMD and MD respectively. All MD outliers are also rMD outliers.

The MD vs rMD plot reveals that the data can be divided into three groups: 1) 155 samples that form the core of the set (Fig 3–gray points), 2) 100 samples that are rMD outliers only (Fig 3–blue points) 3) 22 samples that are outliers according to both MD and rMD (Fig 3 red points marked blue). This shows that the classic MD is more conservative in terms marking outliers than the rMD. Both measures MD and rMD calculate the distance of data points from the data center. However while MD uses all points to determine the data center location, rMD uses only a subset of points that are the closest to the center (see Methods for more details). If a dataset consists of two subsets of points then rMD may use only one of them two determine the center of the data (this depends on the sizes of subsets). In such a situation points from the other set may be seen as outliers in terms of rMD. That is why this measure can be successfully used to state whether the set is homo- or heterogeneous.

### Hierarchical clustering

We performed two rounds of clustering: 1) clustering of attributes–attributes were treated as instances and patients were treated as attributes, 2) clustering of patients—patients were treated as instances and their parameters were treated as attributes.

**Fig 4:**
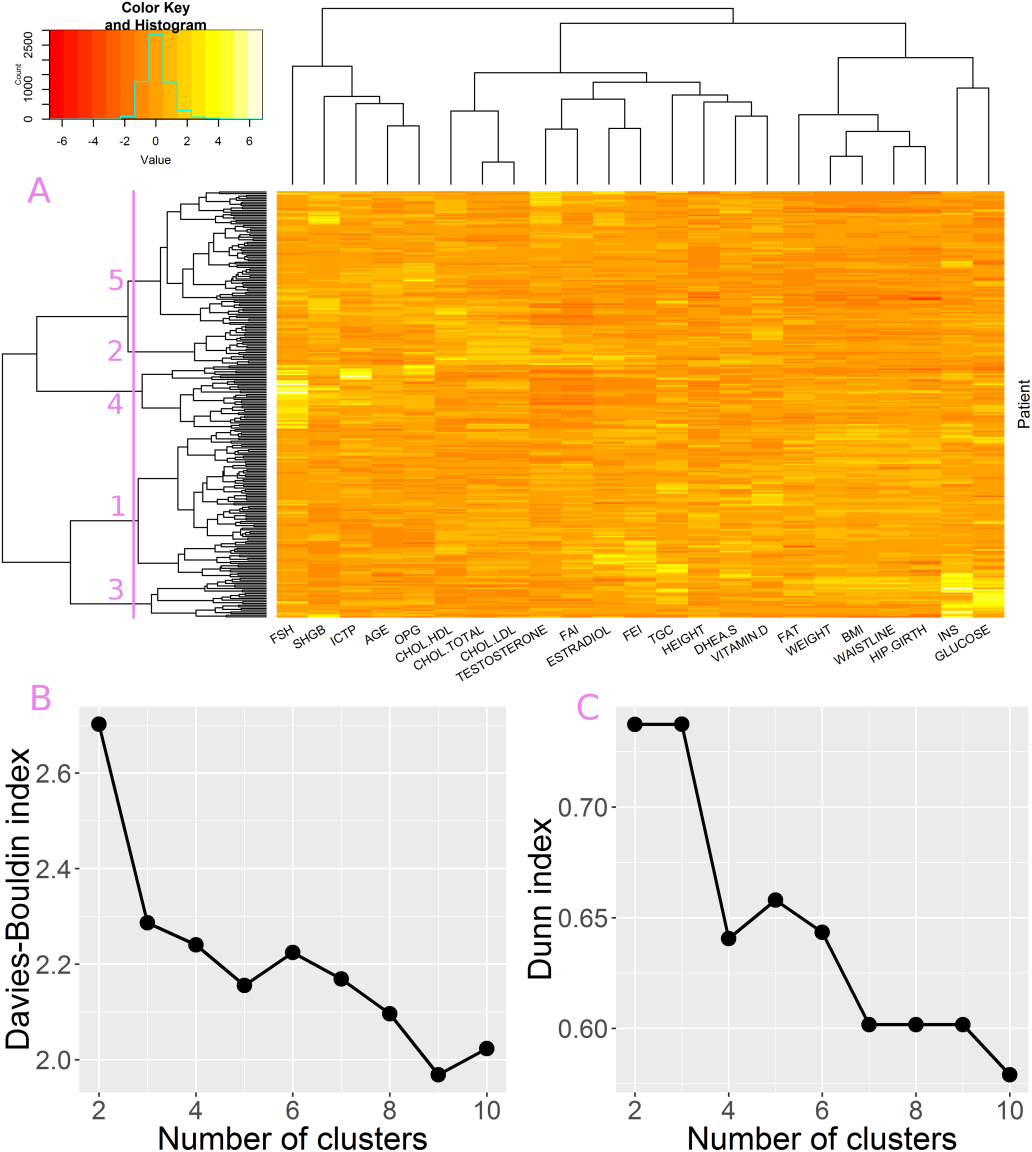
Hierarchical clustering analysis. A) top panel–attribute clustering tree, left panel–patient clustering tree, central panel–dataset heatmap; branch length is proportional to distances between clusters B) Davies Bouldin index for patient partitioning into 2–10 clusters C) Dunn index for patient partitioning into 2–10 clusters.

**Fig 5:**
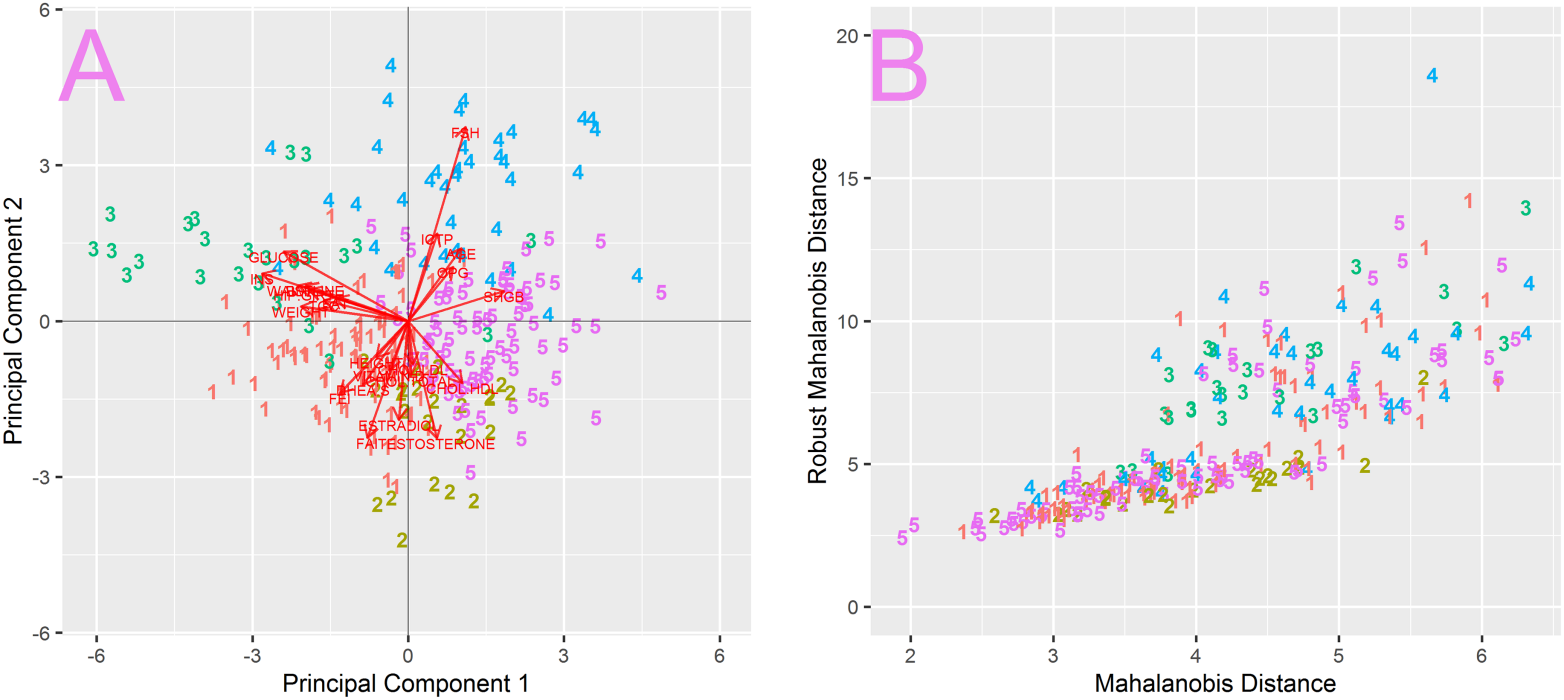
Visualization of clustered male samples. A) in PCA biplot, B) MD vs rMD metrics.

Clustering of attributes showed that there are three main groups of parameters (Fig 4A—top panel), i.e. age-related parameters (FSH, SHGB, ICTP, AGE, OPG), cholesterol and sex-hormone related parameters (including TESTOSTERONE, ESTRADIOL, DHEA), and metabolism related parameters (such as FAT, WEIGHT, BMI, GLUCOSE and INSULINE). This division was also confirmed in the PCA biplot, which depicts three groups of attribute vectors pointing in similar directions (Fig 5A). These three groups correspond well to groups revealed by clustering.

The patient clustering tree is presented in Fig 4A–left panel. Acquired partitioning was validated using Davies-Buildin (DB) and Dunn indices at different tree cut levels, i.e divisions into 2 to 10 clusters were analyzed. Neither DB nor Dunn index clearly indicated which cluster partitioning is the most appropriate (Fig 4B and 4C). In case of the DB good partitioning is indicated by small values. As depicted in Fig 4B, DB index decreases as the number of clusters increases, with a local minimum formed for the division in to 5 groups. In case of the Dunn index a good partitioning is indicated by high values. The highest values can be observed for partitioning into 2 and 3 clusters. However, a local maximum can be observed at the division into 5 groups (Fig 4C). Since both indices emphasized clustering into 5 groups, this partitioning is analyzed in greater details.

Partitioning the set into 5 groups results in two large clusters- cl #1 and cl #5, of 89 and 80 samples respectively and three smaller clusters cl #2–24 samples, cl #3–24 samples and cl #4–38 samples. According to MD and rMD metrics clusters #1, #2 and #5 form the core of the data as shown in Fig 5B, while clusters #3 and #4 deviate from the core and form the majority of RD outliers (Fig 5B).

**Fig 6:**
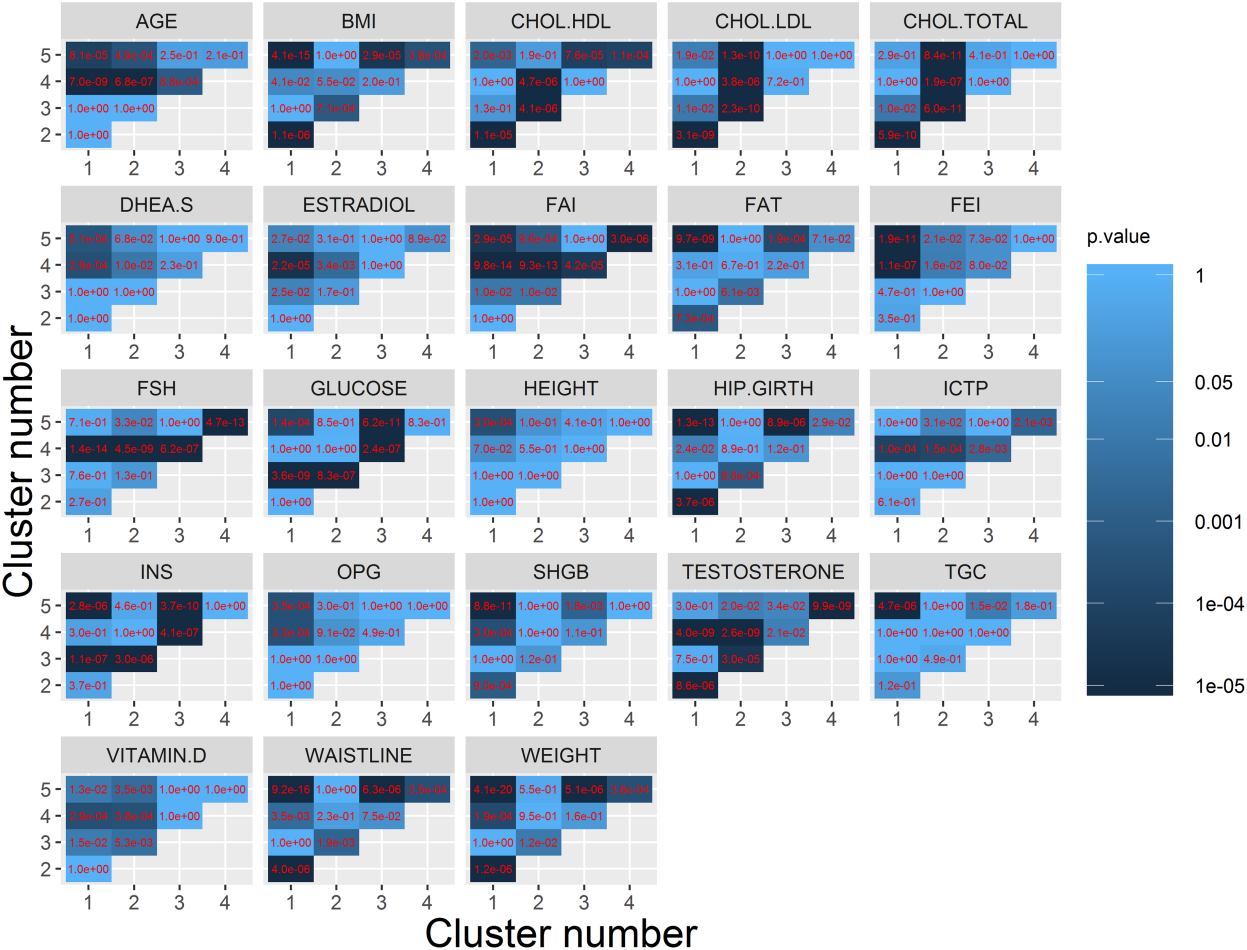
Head-to-head comparisons of attributes between 5 clusters with Wilcoxon rank sum test. Values in red denote p-values.

The significance of differences between all clusters in terms of particular attributes were tested first with the Kruskal-Wallis test [29] and then paired Wilcoxon rank sum test with Bonferroni correction. In Fig 6 p-values of all-vs-all Wilcoxon tests were shown.

Cluster #3 is characterized by significantly elevated levels of INSULINE and GLUCOSE. This is clearly visible in the clustering heatmap as a bright area in INS and GLUCOSE columns (Fig 4A). In PCA bioplot members of the cluster are localized far away from the center of the dataset along INS and GLUCOSE vectors (Fig 5A). The significance of difference between #3 and members of other clusters was confirmed by statistical tests (Fig 6). We suspect this cluster may be a group of putative diabetes patients. Cluster #4 is characterized by exceptionally high levels of FSH and ICTP hormones, which are accompanied by low level TESTOSTERONE and decreased ESTRADIOL. The group is also characterized by greater AGE values. FAI and FEI attributes are also low in this group of patients, however this was expected since TESTOSTERON and FAI as well as ESTRADIOL and FEI are related attributes. In the PCA biplot (Fig 5A) Members of cluster #4 are localized far away from the center of the dataset along the FSH and ICTP vectors. High FSH and low serum level of TESTOSTERONE may indicate that these patients suffer from primary hypogonadism [30].

The core of the data in terms of MD and rMD is formed by clusters #1, #2 and #5. Cluster #2 is the smallest of them. As featured by the dendrogram (Fig 4A–left panel) it is closely related to cluster #5. With the main difference between them being the elevated levels of cholesterol (CHOL.LDL, CHOL.HDL, and CHOL.TOTAL). Members of both clusters are characterized by relatively high TESTOSTERONE levels.

The largest clusters #1 and #5 are hard to be characterized since they form a reference point for describing remaining clusters. The main difference between them comes from metabolism-related attributes: WEIGHT, WAISTLINE, BMI, HIP.GIRTH, FAT, TGC, INS, GLUCOSE. This can be observed in the clustering heat map as a darker patch in the region of cluster #5 (Fig 4A). The difference became more evident after addition of categorical data, which included metabolic phenotype classifications (see next section). The clusters also differ in terms of SHGB and FEI, FAI levels. In the PCA biplot members of cluster #5 are shifted in the opposite direction to the one pointed by metabolic attributes (Fig 5A) and also towards the SHGB direction. The latter confirms higher SGHB values in this cluster. Quite interestingly members of both largest clusters can be found not only in the core of the data but also in the rMD outlier group (Fig 5B), which means that further division might reveal some interpretable subgroups.

### Addition of categorical data

**Fig 7:**
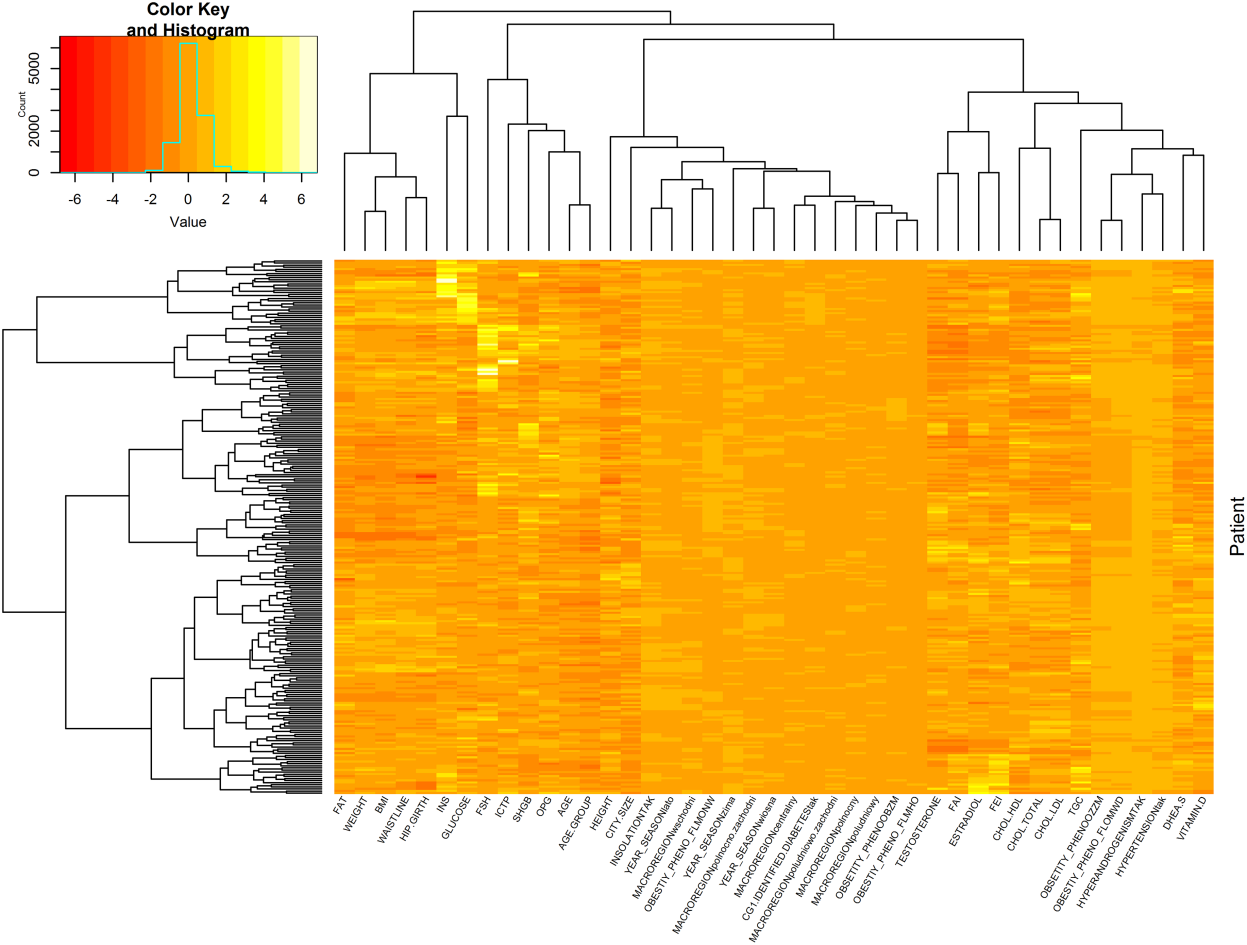
Hierarchical clustering of the male set with categorical and nominal attributes added.

Categorical attributes were transformed to binary attributes and scaled as described in Methods section. Hierarchical clustering with Wards algorithm was repeated. Clustering validation Davies-Bouldin and Dunn indexes both indicated division into three clusters as the most appropriate partitioning (data not shown). Two of the clusters could be easily identified as outlier clusters #3 (aberrant GLUCOSE and INS levels) and #4 (aberrant FSH and ICTP) from the numerical attribute clustering analysis. The third cluster forms the core of the data which includes clusters #1, #2 and #5 (Fig 7—left panel). Obesity phenotype attributes present in in the set of categorical attributes confirmed that the main difference between cluster #1 and clusters #2 and #5 is related to metabolism–dark patch in OBESITY_PHENOOZZM and OBESTITY_PHENO_FLOMWD and light patch in OBESITY_PHENO_FLMONW (Fig 7—heatmap).

### Other clustering approaches

Applying additional methodologically divergent approaches may strengthen the final conclusions or suggest other optional viewpoints. We supported the main clustering analysis with three alternative approaches: density-based DBSCAN clustering, hierarchical clustering based on top principal components and biclustering focused on identification of coherent values. While PC based clustering and biclustering approaches led to conclusions compliant with those already presented, the density based approach was unable to uncover the underlying structure of the data. The majority of samples fell into a single cluster and only a few marginal samples were marked as noise (see S1B Fig). Most probably this is due to the fact that the subgroups overlap and also are characterized by similar point densities, which make them hard to separate by the DBSCAN algorithm. However, the method was successfully applied to support outlier detection. When we ran the algorithm on the dataset containing outliers, the algorithm marked 31 samples as noise. All of them were also marked as outliers by either MD or RD distances (S1 Table).

Opposite to DBSCAN clustering–the clustering based on top 7 PCs, which accounted for 70% of data variance, resulted in a partitioning very similar to the one acquired by the main clustering approach (S2 Fig).

Finally, the main conclusions were also supported by the outcome of the biclustering plaid model analysis. All significant clusters and relations were found. However, the clusters were smaller and the outcomes were subject to some the randomness due to the nature of the clustering algorithm (S3A–S3D Fig).

### Female set analysis

The female set was analyzed using the same methodology that was applied in male set analysis. The set included 238 patients with 23 numeric attributes. Data were normalized with the Z-score robust normalization, then outlier analysis was carried out with MD and robust MD distances, finally we performed hierarchical clustering analysis supported with DB and Dunn clustering validation indices.

**Fig 8:**
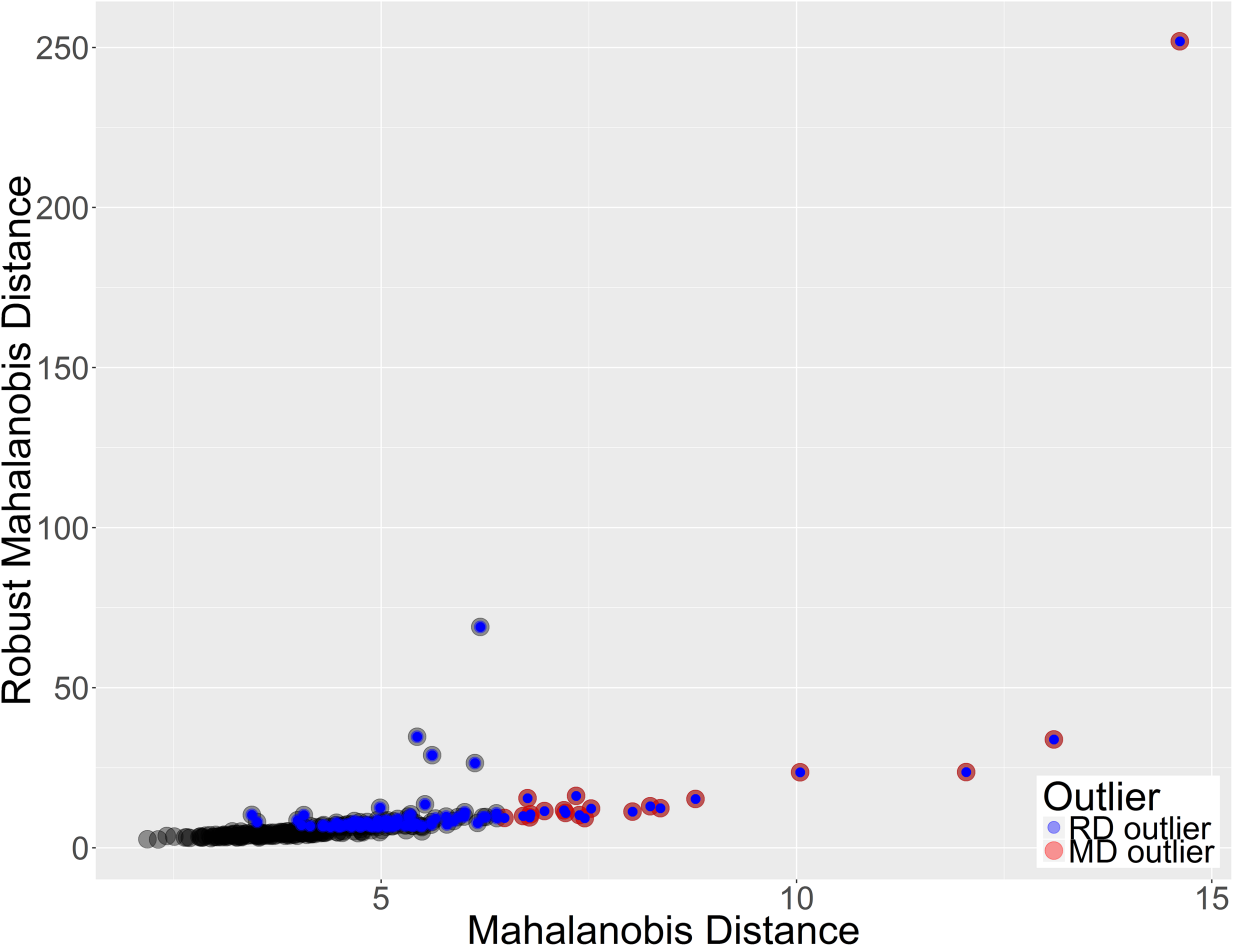
Outlier analysis in the female set. MD and rMD are consistent–most points lie on a straight line.

Outlier analysis in the female set indicates 70 and 20 robust MD and MD outliers respectively. All MD outliers were also robust MD outliers. The robust MD vs MD plot differs significantly from the plot acquired in the male set analysis–points are more condensed and cannot easily divided into subgroups (Fig 8). Although there are many outliers according to rMD, it seems that only a few of them are actual outliers. The majority of rMD outliers remain quite close to the core of the dataset in terms of MD. This suggests that female dataset is more homogeneous than the male dataset.

**Fig 9:**
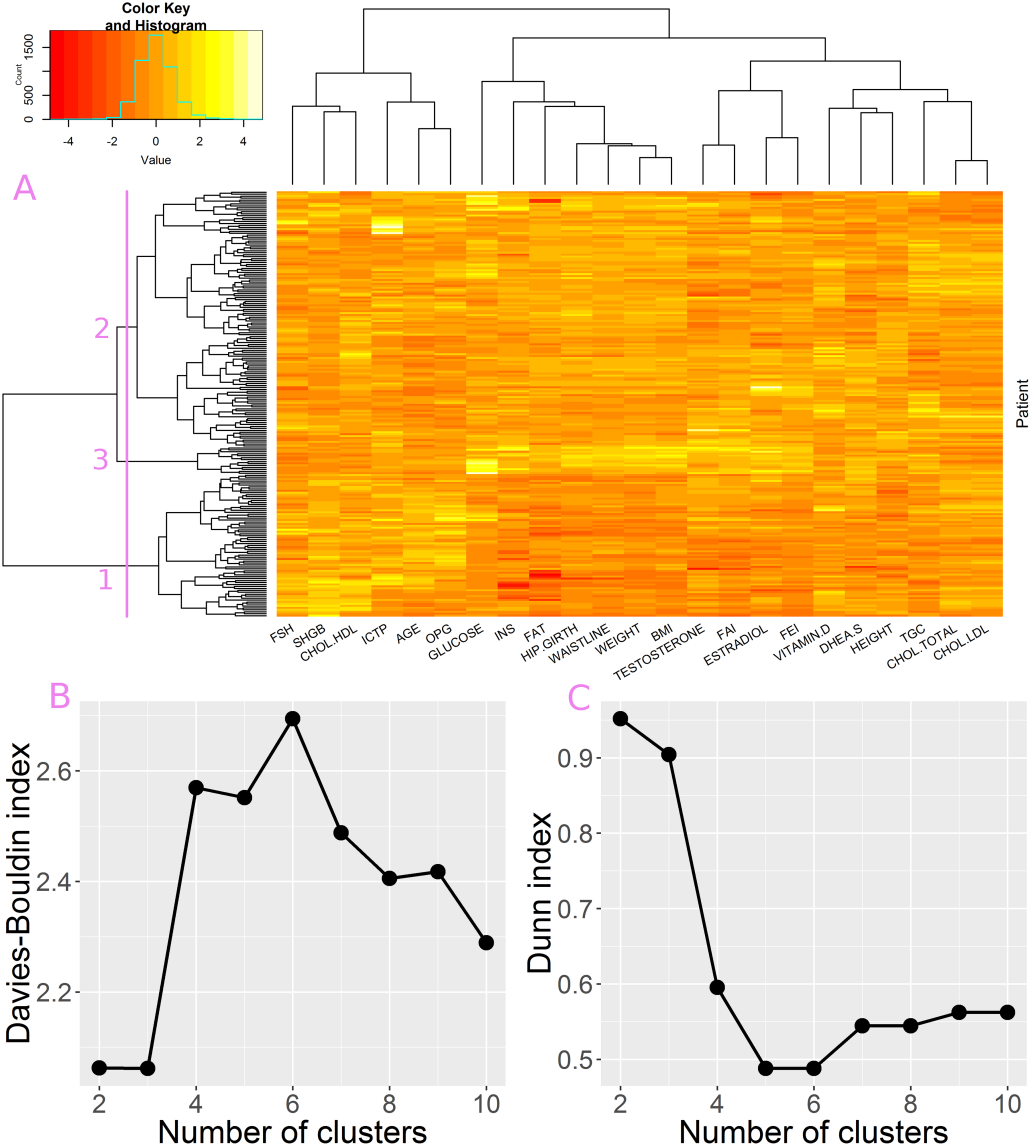
Clustering analysis in the female dataset. (A) The violet line and labels on the dendrogram denote the best partitioning according to cluster validation indices. Davies- Bouldin index (B) and Dunn index(C) indicate that partitioning the set into 2 or 3 clusters are the best choices for further analysis (low DB and high Dunn values).

Hierarchical clustering of attributes confirmed the division revealed in male set analysis, i.e. three attribute groups were identified: age-related parameters (FSH, SHGB, ICTP, AGE, OPG), cholesterol and sex-hormone related parameters (including TESTOSTERONE, ESTRADIOL, DHEA), and metabolism related parameters (Fig 9A–top panel). The HDL Cholesterol level was an exception–in this analysis it is part of the age related attribute group.

According to DB and Dunn indices the optimal division of female patients includes two or three groups (Fig 9B and 9C). We analyzed the three cluster division as it is more informative. In this case cluster #1 consists of 71 patients. These patients are characterized by low values of metabolic parameters (Fig 9A–heatmap, dark path in GLUCOSE, INS, FAT and others), and elevated levels of SHGB, FSH, CHOL.HDL. Cluster #2 groups 131 patients. It forms the core of the dataset and probably represents the majority of population. Finally cluster #3, a cluster of 14 patients with high levels of metabolic parameters (GLUCOSE, INS, FAT and others) but also elevated levels of TESTOSTERONE and ESTRADIOL.

The biplot visualization of the data is consistent with both: clustering of attributes and clustering of patients. The contributions of particular attributes in PCs confirm the relations between parameters–metabolic parameters and hormone related parameters form two well distinguishable groups of similarly pointing vectors. The third group is more diverse, but the sub groups are correct, i.e OPG, AGE and ICTP form one group and FSH SHGB and CHOL.HDL form a second group of vectors (Fig 9 red arrows). The distribution of patients in the biplot is also consistent with the clustering. Members of cluster #1 are localized in the region pointed by SHGB, FSH and CHOL.HDL vectors, and opposite the direction of metabolic attributes. Members of cluster #2 are in the center of the plot, while members of cluster #3 are shifted away from the origin mainly in the direction of metabolic attributes.

**Fig 10:**
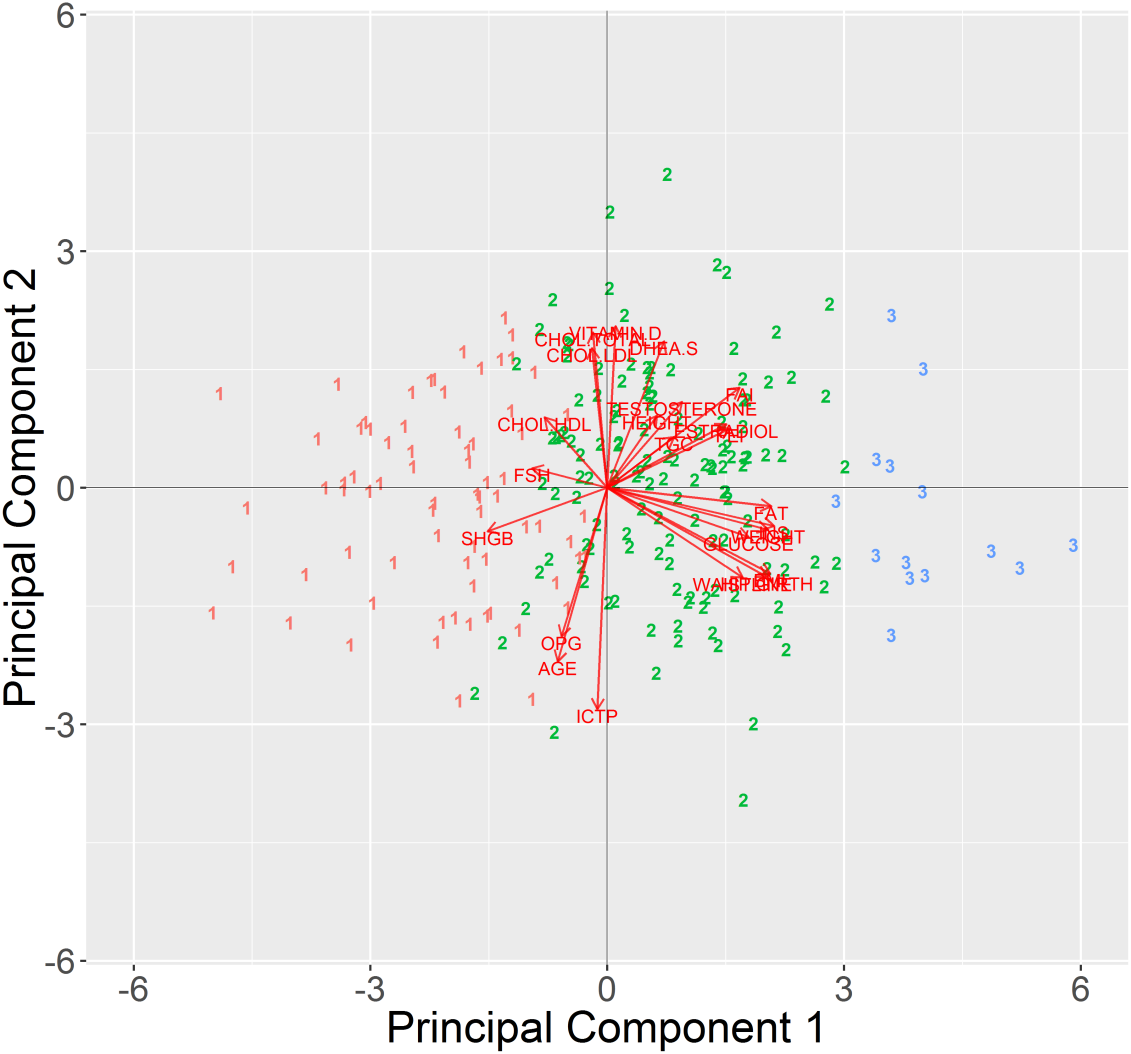
PCA biplot of the female dataset. Patients form a quite condensed cloud of point (we just a few exceptions). The clusters result from natural biological variation rather than from pathologies.

Over all the PCA plot of the female set is more homogeneous in comparison to the PCA in the male set analysis (Fig 10). Samples present are more evenly distributed around the origin, while in the male set subgroups could be easily distinguished. This suggests that in the female set there are no pathological groups of patients that could be recognized based on the set of attributes at hand. However still, there are some patients that should be investigated and verified prior to including them in further studies (for instance three patients in cluster #3 furthest away from the origin).

## Conclusion

In this work we presented a data exploratory analysis of a clinical study group. Each patient was described by over 40 numerical and nominal attributes. The aim of the study was to reveal the structure of the data, i.e. verify whether the population of patients is homogenous or whether subpopulations are present. We also wanted to characterize identified subgroups and to investigate basic relations between attributes. The analysis was performed with a set of methods that were specially selected to work well together. First a robust normalization technique was used. Then MD based outlier detection methods, hierarchical clustering with Wards algorithm and PCA visualization was performed. Since all these methods take in to account the correlation and variance of data attributes, their outcomes were consistent. We have shown that the MD/rMD analysis allows not only to identify outliers but can also be used to assess the heterogeneity of a dataset. PCA together with the biplot allowed to characterize data instances and explain the acquired clustering. The analysis was additionally supported by three alternative clustering approaches, which strengthen the main conclusions and contributed to better understanding of the data.

Several important biological conclusion can be drawn. The study showed significant differences between male and female patients. In the male set we managed to identify five distinct patient groups, two of which were recognized as clusters of putatively diseased patients. In further analysis this structure should be taken into account. One should consider testing scientific hypothesis separately in each of identified subgroups. Depending on the aims of subsequent investigation some of the groups should be removed or treated in a special way.

The female set was more homogenous in comparison to the male set and the clusters we identified were not recognized as pathological. However, still one might also consider performing further investigations separately in the identified subgroups.

Neglecting the fact of existence of patient subgroups might make it impossible to reveal important biological phenomena or in the worst case lead to false conclusions.

## Supporting information

S1 Table Analysis data. (CSV)

S2 Table Comparison of sample labeling by density-based clustering and Mahalanobis Distances. (DOCX)

S1 Code Exploratory analysis code. (R)

S1 Fig Density based clustering on the male dataset. A) the parameters chosen for clustering were K = 3 neighbors and epsilon = 4 (based on the elbow method), B) density clustering failed to confirm the structure of the data revealed by hierarchical clustering by managed to mark marginal points (zero’s) and could be used for outlier detection.
(TIFF)

S2 Fig Hierarchical clustering based on first 7 Principal Components shows high accordance with clustering based on full attribute set. Most importantly clusters of patients with high levels of FSH or GLUCOSE/INSULIN were found (blue and green cluster respectively).
(TIFF)

S3 Fig Subclusters identified by plaid model biclustering. The analysis resulted in identifying the two important outlier clusters: A) the cluster with elevated INSULIN and GLUCOSE levels and B) patients with elevated FSH levels. In addition two other patient subgroups were found: C) one showing a dependence of hormone and cholesterol related attributes and D) group of patients with simultaneously elevated SHGB and CHOL.HDL levels.
(TIFF)
