# Supplementary material for: Exploratory data analysis of a clinical study group: Development of a procedure for exploring multidimensional data
Source: PLoS One. 2018 Aug 23;13(8):e0201950. doi: 10.1371/journal.pone.0201950 (PMC6107146; doi:10.1371/journal.pone.0201950)
Supplement: S2 Table — (DOCX) [file pone.0201950.s002.docx]

**S2 Table.** Comparison of sample labeling by density-based clustering and Mahalanobis Distances

| **Density Based clusters** | **Non-outlier** | **RD-only outlier** | **MD-only outlier** | **MD & RD outlier** | **TOTAL** |
| --- | --- | --- | --- | --- | --- |
| Cluster 0 | 0 | 14 | 0 | 17 | **31** |
| Cluster 1 | 153 | 88 | 0 | 5 | 246 |
