# Supplementary figures and images for: Exploratory data analysis of a clinical study group: Development of a procedure for exploring multidimensional data
Source: PLoS One. 2018 Aug 23;13(8):e0201950. doi: 10.1371/journal.pone.0201950 (PMC6107146; doi:10.1371/journal.pone.0201950)

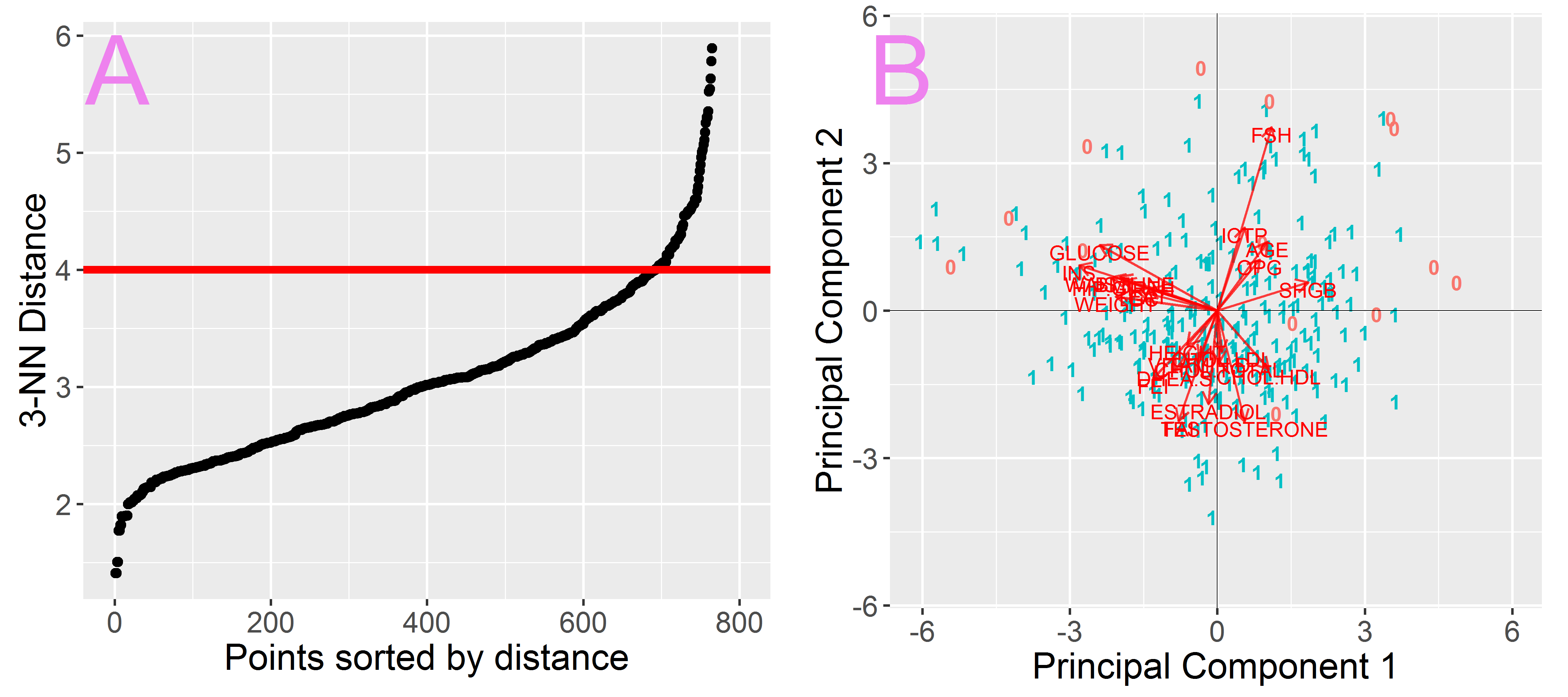

Supplement: S1 Fig — A) the parameters chosen for clustering were K = 3 neighbors and epsilon = 4 (based on the elbow method), B) density clustering failed to confirm the structure of the data revealed by hierarchical clustering by managed to mark marginal points (zero’s) and could be used for outlier detection. (TIFF) [file pone.0201950.s004.tiff]

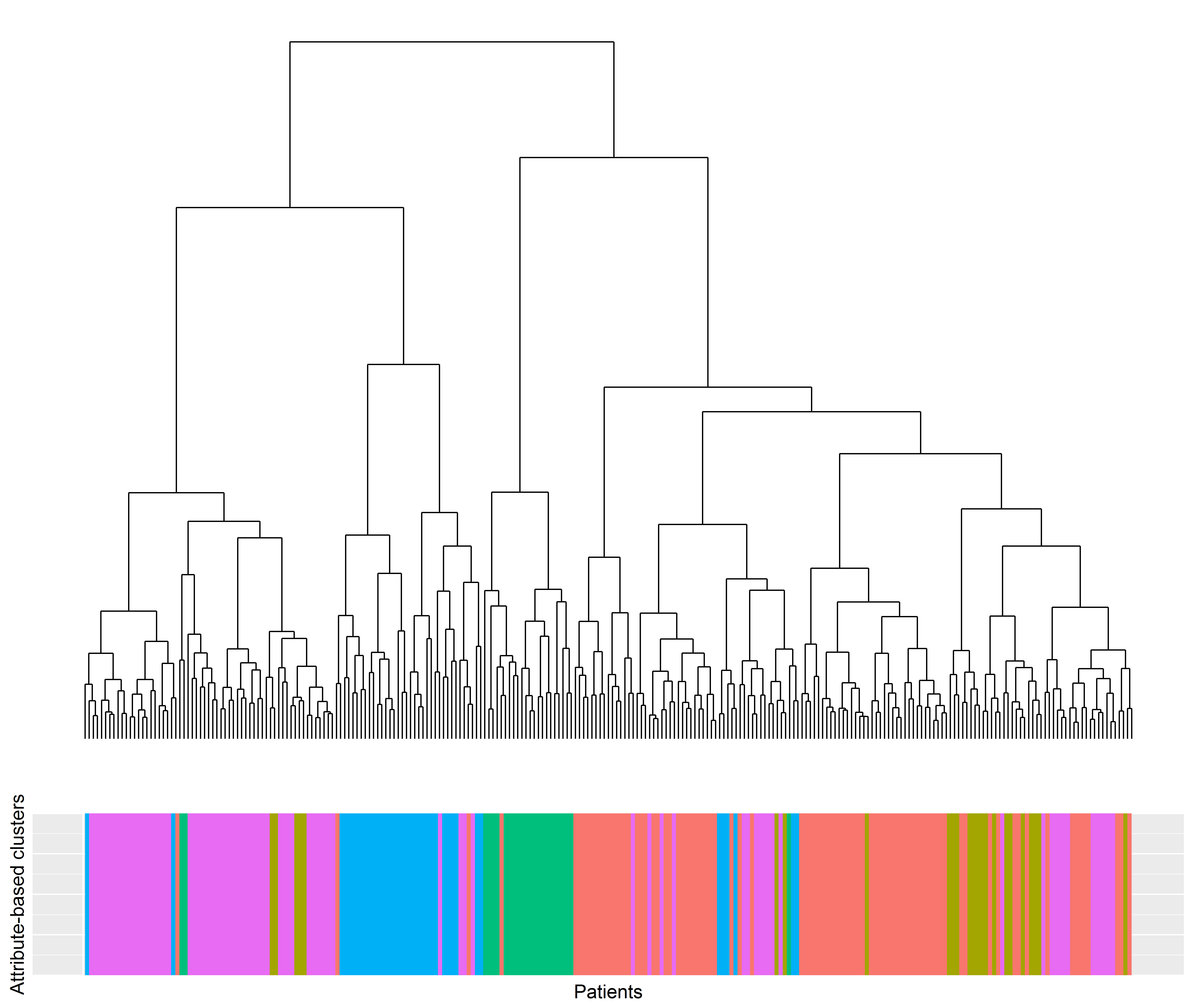

Supplement: S2 Fig — Most importantly clusters of patients with high levels of FSH or GLUCOSE/INSULIN were found (blue and green cluster respectively). (TIFF) [file pone.0201950.s005.tiff]

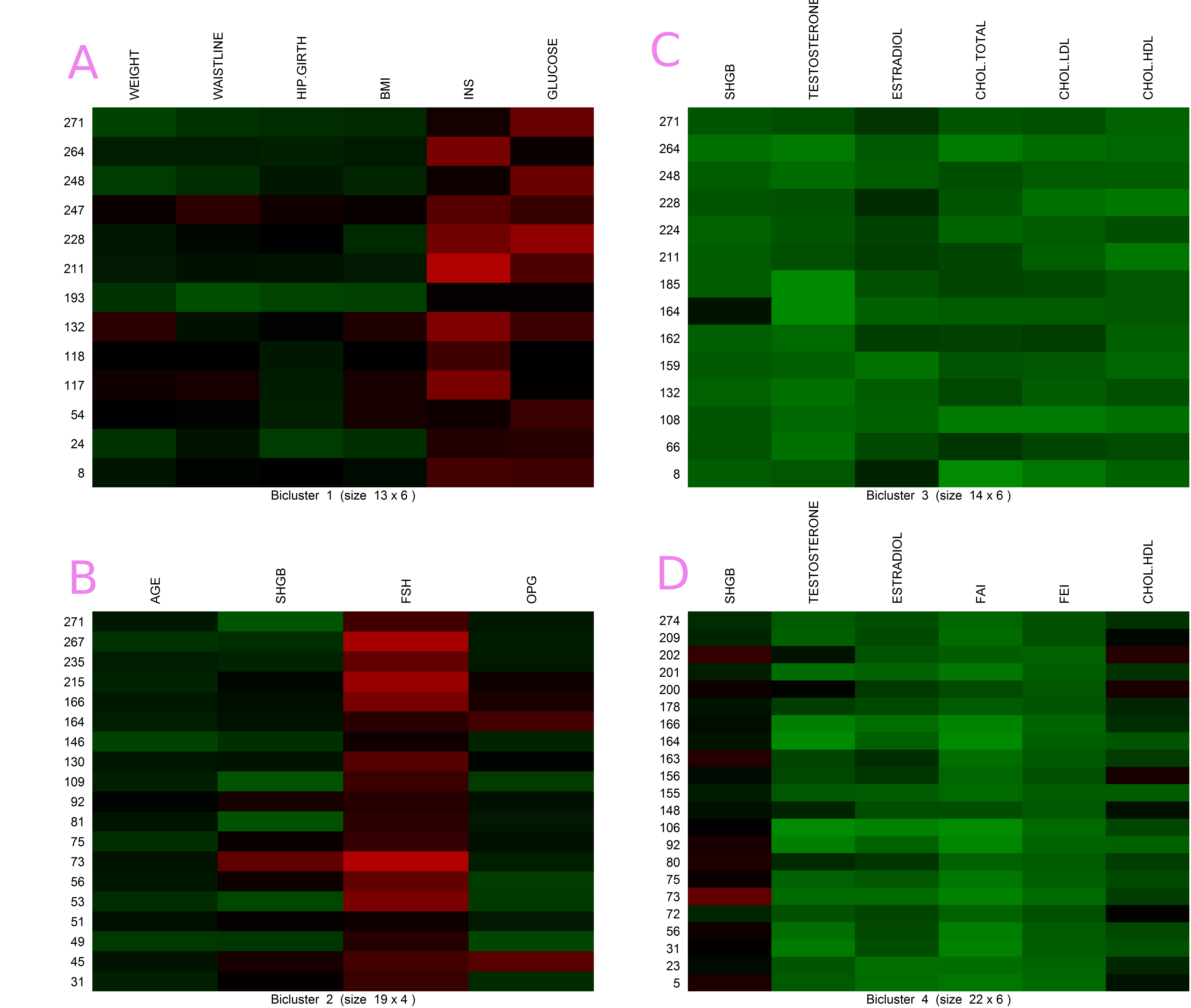

Supplement: S3 Fig — The analysis resulted in identifying the two important outlier clusters: A) the cluster with elevated INSULIN and GLUCOSE levels and B) patients with elevated FSH levels. In addition two other patient subgroups were found: C) one showing a dependence of hormone and cholesterol related attributes and D) group of patients with simultaneously elevated SHGB and CHOL.HDL levels. (TIFF) [file pone.0201950.s006.tiff]
